# Learning multiple rules simultaneously: Affixes are more salient than reduplications

**DOI:** 10.3758/s13421-016-0669-9

**Published:** 2016-11-21

**Authors:** Judit Gervain, Ansgar D. Endress

**Affiliations:** 1 Laboratoire Psychologie de la Perception, CNRS, 45 rue des Saints-Pères, Paris, 75006 France; 2 Laboratoire Psychologie de la Perception, Université Paris Descartes, Sorbonne Paris Cité, 45 rue des Saints-Pères, Paris, 75006 France; 3 Department of Psychology, City, University of London, Northampton Square, London, EC1V 0HB UK

**Keywords:** Perceptual or memory primitives, Bayesian learning, Rule-learning, Artificial grammar learning, Edges

## Abstract

Language learners encounter numerous opportunities to learn regularities, but need to decide which of these regularities to learn, because some are not productive in their native language. Here, we present an account of rule learning based on perceptual and memory primitives (Endress, Dehaene-Lambertz, & Mehler, *Cognition*, **105**(3), 577–614, 2007; Endress, Nespor, & Mehler, *Trends in Cognitive Sciences*, **13**(8), 348–353, 2009), suggesting that learners preferentially learn regularities that are more salient to them, and that the pattern of salience reflects the frequency of language features across languages. We contrast this view with previous artificial grammar learning research, which suggests that infants “choose” the regularities they learn based on rational, Bayesian criteria (Frank & Tenenbaum, *Cognition*, **120**(3), 360–371, 2013; Gerken, *Cognition*, **98**(3)B67–B74, 2006, *Cognition*, **115**(2), 362–366, 2010). In our experiments, adult participants listened to syllable strings starting with a syllable reduplication and always ending with the same “affix” syllable, or to syllable strings starting with this “affix” syllable and ending with the “reduplication”. Both affixation and reduplication are frequently used for morphological marking across languages. We find three crucial results. First, participants learned both regularities simultaneously. Second, affixation regularities seemed easier to learn than reduplication regularities. Third, regularities in sequence offsets were easier to learn than regularities at sequence onsets. We show that these results are inconsistent with previous Bayesian rule learning models, but mesh well with the perceptual or memory primitives view. Further, we show that the pattern of salience revealed in our experiments reflects the distribution of regularities across languages. Ease of acquisition might thus be one determinant of the frequency of regularities across languages.

## Introduction

Acquiring language involves learning multiple regularities about the internal structures of linguistic units, such as words, phrases and sentences. These regularities can apply to different properties of linguistic units, for instance their identity, their position and the relations between them, and, more often than not, multiple regularities apply to any given linguistic object. For example, a word in a sentence conforms to regularities about its sound structure, its intonation, its morphology, its relation to other words, the social and pragmatic context of the sentences and so on. Further, the regularities can also differ in their scope, some applying to only a few items, others to entire categories of items, with or without exceptions. Given these complexities, it is nothing short of astounding that infants manage to become competent speakers of their native language.

Understanding how learners extract grammatical regularities from speech has been the focus of much research in cognitive science in the last decades (e.g., Christophe, Nespor, Guasti, & Van Ooyen, 2003; Endress, Nespor, & Mehler, 2009; Gervain, Nespor, Mazuka, Horie, & Mehler, 2008; Kovács & Endress, 2014; Lidz, Gleitman, & Gleitman, 2003; Morgan, 1986; Morgan & Demuth, 1996; Onnis, Waterfall, & Edelman, 2008; Peña, Bonatti, Nespor, & Mehler, 2002; Saffran & Wilson, 2003; Toro, Bonatti, Nespor, & Mehler, 2008). However, we still do not have a full account of the acquisition of grammar, and we know even less about how learning proceeds when learners are faced with several regularities simultaneously.

In one of the few studies on this question, Gerken (2006) presented infants with syllable triplets that conformed to two regularities. First, the first two syllables of each triplet were repeated. Second, all triplets ended with /*di*/, yielding triplets like /leledi/. We will refer to these patterns as AA/*di*/ patterns. (Other infants were presented with an A/*di*/A pattern, where the first and the last syllable were identical, and the middle syllable was /*di*/. For ease of exposure, we will gloss over these conditions).

Although both humans and non-human animals can learn repetition-patterns (for humans: see e.g., Gervain, Macagno, Cogoi, Peña, & Mehler, 2008; Kovács & Mehler, 2009; Marcus, Vijayan, Rao, & Vishton, 1999; Saffran, Pollak, Seibel, & Shkolnik, 2007; for non-human animals: see e.g., Giurfa, Zhang, Jenett, Menzel, & Srinivasan, 2001; Hauser & Glynn, 2009; Martinho & Kacelnik, 2016; van Heijningen, Chen, van Laatum, van der Hulst, & ten Cate, 2013) and regularities about the first and the last position of sequences (for humans: see e.g., Endress & Wood, 2011; Seidl & Johnson, 2006; Gervain, Berent, & Werker, 2012; for non-human animals: Chen, Jansen, & Ten Cate, 2016; Endress, Cahill, Block, Watumull, & Hauser, 2009), infants appeared to learn only one of the regularities in the Gerken (2006) study: they learned only that triplets had to end in /*di*/.

Do such results imply that people can learn only one regularity at a time? This possibility seems unlikely, because, in some situations, both adults and infants do learn multiple regularities at the same time (e.g., Endress & Bonatti, 2007; Endress & Wood, 2011; Marchetto & Bonatti, 2013; Peña et al., 2002). Gerken (2010) tested this issue by first familiarizing infants to the AA/*di*/ (or A/*di*/A) pattern as described above. Crucially, however, infants were then presented with three examples of an AAB pattern (where the last syllable was no longer /*di*/), intermixed with the last five familiarization stimuli. Under these conditions, infants learned the repetition-pattern. In a critical control condition, Gerken (2010) asked whether infants learned the repetition-pattern just based on the last five examples, and replaced the AA/*di*/ familiarization with music. Results showed that, under these conditions, infants did not learn the repetition-pattern.

Together, these results thus suggest that infants have a trace of the repetition-pattern also when familiarized with an AA/*di*/ pattern; however, they will show generalization only if also familiarized with items that do not conform to the /*di*/ pattern. Gerken (2010) suggested that infants use rational decision criteria for their generalizations, and make the narrowest possible generalization that is compatible with the familiarization.

### Bayesian approaches to rule learning

Frank and Tenenbaum (2013) formalized this idea using a Bayesian model. Specifically, with *S* syllables, one can form *S* ^2^ triplets that end in /*di*/ (or (*S*−1)^2^ triplets if the first two syllables cannot be /*di*/). Likewise, one can form *S* ^2^ triplets where the first two syllables are identical (or *S*(*S*−1) if the last one has to be different from the first two). Thus, considered separately, the two rules allow for equally broad generalizations. However, Frank and Tenenbaum (2013) proposes that infants do not only represent these two atomic patterns, but also a conjunction pattern where the first two syllables are repeated *and* the last one is /*di*/. One can form *S* such triplets (or *S*−1 if the first two syllables cannot be /*di*/). Hence, the conjunction pattern generates fewer potential triplets. Following Tenenbaum and Griffiths (2001), infants should thus choose the conjunction pattern, as it provides the narrowest possible generalization. This is called the size principle, and is a frequent assumption in Bayesian models of cognition (see, among many others, Denison, Reed, & Xu, 2013; Gweon, Tenenbaum, & Schulz, 2010; Navarro, Dry, & Lee, 2012; Xu & Tenenbaum, 2007a, 2007b). This conjunction pattern is important, because it is at the root of Frank and Tenenbaum’s (2013) model’s success.

This model represents a tradition where learners — explicitly or implicitly — “optimize” what they learn from examples. An alternative view is that some rules might be learned by perceptual or memory primitives (e.g., Endress et al., 2007; Endress, Scholl, & Mehler, 2005; Endress, Nespor, & Mehler, 2009). According to the latter view, some rules just pop out by their salience, and we learn whatever is salient to us.

### The perceptual primitives approach to rule-learning

In line with the latter view, Endress (2013) proposed an alternative account for the aforementioned data. He made three main hypotheses. First, repetitions and items in edges of sequences are tracked by independent mechanisms, the former by some kind of repetition-detector and the latter by processes of serial memory (Endress & Mehler, 2009). As a result, infants might not represent a conjunction rule; they might just notice that items end in /*di*/ and start with a repetition. In other words, the regularities to which a string conforms might essentially be treated as features of that string.

Second, infants expect items to conform to all generalizations they have picked up (see Gerken, Dawson, Chatila, & Tenenbaum, 2015, for an empirical confirmation of this point). As a result, they might consider triplets as a violation if *any* of the rules is violated. For example, when familiarized with AAB triplets (where the last syllable is not systematically /*di*/), infants should be sensitive to violations of the repetition-pattern, because this is the only regularity present in the data. In contrast, when familiarized with AA/*di*/ triplets, both AAB and ABB triplets are violations, since they do not conform to the /*di*/ regularity. Third, some generalizations are more salient than others, and might be more likely to drive behavior. For example, if the /*di*/ regularity is more salient than the repetition pattern, infants might accept violations of the repetition-pattern as long as the /*di*/ regularity is respected.

If this account is correct, the role of the five additional familiarization triplets in Gerken’s (2010) studies might be to familiarize infants with items not containing /*di*/, which, in turn, would allow them to reveal their learning of the repetition-pattern in the subsequent test phase without showing surprise at triplets not containing /*di*/.

### Predictions of the Bayesian and the perceptual primitives approaches

Here, we investigate under what conditions simple rules can be learned, and more specifically test the aforementioned views on rule learning. The Bayesian accounts above differ from Endress’s (2013) model in two key predictions.

First, if infants choose the narrowest possible generalization, they have no reason to prefer the /*di*/ pattern over the repetition pattern; as mentioned above, the number of potential triplets conforming to these patterns is identical. In contrast, Endress (2013) specifically proposes that some patterns might be more salient than others, for no obvious formal reason but just as a consequence of how our mental apparatus happens to have evolved.^1^ Intuitively, one could expect that the /*di*/ regularity might be easier to process than the repetition pattern, because it involves a single item, while repetitions involve, among other things, some mechanisms that compare two items.

Importantly, this intuition cannot be justified by formal considerations that do not depend on other assumptions about our mental architecture. More generally, formal considerations are often poor guides to estimating the relative complexity of two cognitive operations. For example, dividing numbers is hard for humans but easy for a computer, while spatial rotations are relatively easy for humans but require substantial computing power in a computer (see Endress et al., 2007, for discussion).

Second, the Bayesian accounts crucially rely on the existence of a conjunction rule to explain the infant data, as it is only the conjunction rule, and not either of the single rules alone, that produce fewer triplets, i.e. a narrower generalization. But what does it mean for two simple rules to be joined into a conjunction rule? The predictions of this assumption are somewhat unclear. Formally speaking, the truth conditions of conjunction (‘and’) require learners to reject items as soon as any of the patterns they picked up is violated. After all, violating either the /*di*/ regularity or the repetition pattern violates the conjunction rule as well. Hence, if learners represent a conjunction rule and preferentially learn the narrowest generalization and discard other generalizations, they should show a binary response pattern, accepting only items that conform to both rules, and equally rejecting items that violate either or both of the component rules.

It is not inconceivable in this framework to predict that there might be a gap between the rejection rate for items that violate both regularities and items that violate only one, and, in fact, Frank and Tenenbaum’s (2013) model predicts just such a gap, at least with certain analyses.

Be that as it might, under the perceptual or memory primitives view, things like item repetitions and items in edges are independent features of strings. As a result, Endress’s (2013) account predicts a more graded response profile, with learners accepting items that conform to both rules, rejecting items that violate both rules, and showing an intermediate response for items that violate only one of the rules; further, learners should be more likely to reject items that violate the more salient rule.

### The current research

Here, we explore these issues in a population of adult learners. We test adults because the larger sample size and larger number of test items that can be used with this population make it inherently easier to reveal graded responses in adults than in infants. To make the experiments somewhat more challenging, we used longer strings than those employed in Gerken’s (2006) study with infants.

Specifically, we ask how two regularities similar to those used by Gerken (2006) are learned simultaneously under different conditions. One regularity concerns the presence and the serial position of a constant syllable (i.e., /*di*/) in 6-syllable-long sequences generated by an artificial grammar. The other regularity concerns the presence and the serial position of a syllable repetition in the same artificial grammar sequences.

**Fig. 1:**
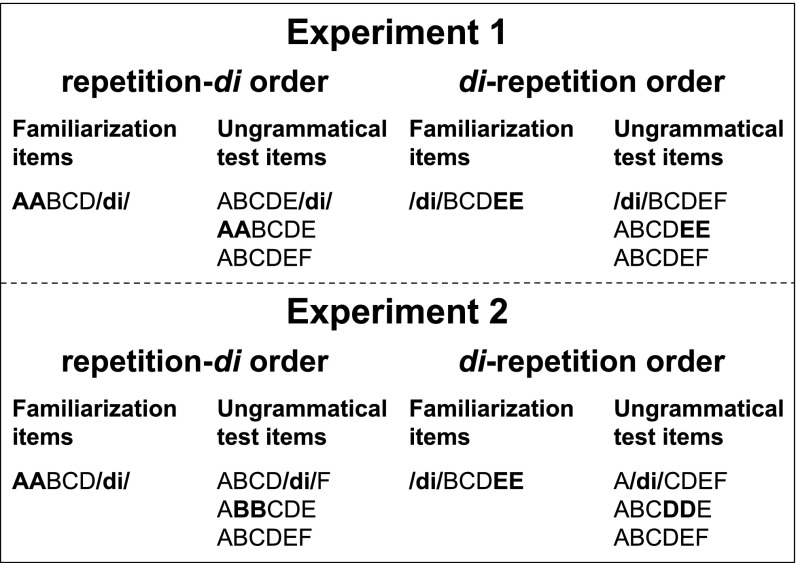
Design of Experiments 1 and 2

The design of the experiments is shown in Fig. 1. In Experiment 1, we test the relative complexity of detecting violations of the presence of either regularity or both. That is, ungrammatical test items did not contain /*di*/, a repetition, or either regularity. In Experiment 2, we tested the saliency of violations of the sequential position of either regularity or both. That is, all ungrammatical test items did contain both /*di*/ and a repetition, but /*di*/, the repetition or both were located in incorrect sequential positions. As a comparison to human performance, we evaluated the predictions of different versions of Frank and Tenenbaum’s (2013) Bayesian model of rule learning.

## Experiments

### Experiment 1: Violation of presence

#### Methods

##### Participants

Participants were 40 monolingual native English-speaking adults (30 females, 10 males, mean age: 20.8 years, range: 18-30 years), recruited at the University of British Columbia, Vancouver, Canada, for course credit.^2^ Participants reported no history of neurological, language or hearing impairment. Participants were randomly assigned to the two grammar conditions (see below), with half of the participants taking part in either condition (‘di-repetition’ or ‘repetition-di’, depending on the relative order of the two repetitions in the sequence).

##### Stimuli

Two artificial grammars generating six-syllable long sequences were created to be used in the familiarization phase of the study. In strings generated by the ‘di-repetition’ grammar, sequences started with the constant syllable /*di*/ and ended with an immediate repetition of a syllable, yielding strings of the form /*di*/ABCDD, where A, B, C and D represent CV syllables. Strings generated by the ‘repetition-di’ grammar started with an immediately repeated syllable and ended in /*di*/, yielding strings of the form AABCD/*di*/.

For the familiarization sequences, categories A and B used possible combinations of the consonants /m/, /n/, /l/, /r/, /p/ and /g/ with the vowels/diphthongs /ei/, /ai/, /ɔi/, and /oʊ/. Categories C and D used the consonants /f/, /v/, /s/, /z/, /b/, and /k/ with the vowels /ɑ/, /ʊ/, /o/, and /aʊ/.

For the test sequences, the consonants were exchanged between the categories such that categories A and B used the consonants /f/, /v/, /s/, /z/, /b/, and /k/ with the vowels /ei/, /ai/, /ɔi/, and /oʊ/, while categories C and D used consonants /m/, /n/, /l/, /r/, /p/ and /g/ with the vowels /ɑ/, /ʊ/, /o/, and /aʊ/. Both for familiarization and test, the sequences were created in such a way that the A and B syllables within the same word always used both different Cs and different Vs to ensure discriminability. The same constraint was applied to D and E syllables within the same word.

For familiarization, 36 sequences were generated for each order, e.g. /*di*/ABCDD: /digɔipeikobɑbɑ/, /digeilaikɑsʊsʊ/, /dirɔilaizosɑsɑ/; AABCD/*di*/: /fɑfɑvomeinɔidi/, /vʊvʊfɑnaimeidi/, /nɔnɔigoʊvokaʊkaʊdi/.

For test, novel grammatical and ungrammatical sequences were created. The grammatical sequences were just like the familiarization sequences, except that they used novel syllables. The ungrammatical sequences either did not contain the syllable /*di*/, did not contain a repeated syllable, or contained neither the syllable /*di*/ nor a repetition. In the following, we will call these kinds of violations violations of *presence*, because the regularities are not present in the strings. This resulted in four types of test items: (i) grammatical items (/*di*/ABCDD or AABCD/*di*/, depending on the grammar a participant had been familiarized with), (ii) repetition violations (/*di*/ABCDE or ABCDE/*di*/), (iii) /*di*/ violations (EABCDD or AABCDE), and (iv) violations of both the repetition and di (ABCDEF). The additional E and F foil syllables needed for the ungrammatical items were randomly chosen from the A, B, C, and D categories in a counterbalanced fashion, making sure that a category is not inadvertently directly repeated as a result, e.g. a syllable from category D was never used as a category E syllable. For each test item type, 9 items were created, for a total of 36 test items per condition.

The sequences were synthesized using the us3 voice of the MBROLA text-to-speech synthesizer (Dutoit, 1997). Each phoneme was 116 ms long, resulting in sequences of 1.392 s. The sequences had a monotonous pitch of 135 Hz.

##### Procedure

Participants were tested individually in a quiet room, seated in front of a computer that delivered the stimuli and recorded participants’ responses. Sound stimuli were presented through high-quality headphones. Participants were informed that they would first listen to a sample of an unknown language (“Martian”), and would then be tested on their knowledge of the ‘sentences’ of the language. Following this, participants were instructed to simply listen to the familiarization sentences.

The familiarization consisted of 36 sentences separated by an inter-stimulus interval of 1 s, presented in a different pseudo-random order for each participant. The familiarization lasted 1 min 44 s. After familiarization, participants passed immediately onto the test phase. In each of the 36 test trials, they heard a novel sentence, and they had to indicate whether it was a Martian sentence. Responses were collected from two predefined keys. No feedback was given after the test trials.

Among the 36 test items, 9 were grammatical, respecting both regularities, 9 violated the repetition regularity, 9 violated the *di* regularity and 9 violated both regularities. The order of presentation of the 36 test items was randomized for each participant with the constraint that no more than three items from the same item type could occur consecutively.

#### Results

**Fig. 2:**
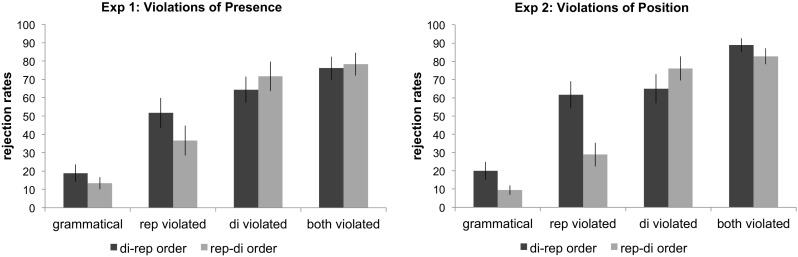
Average rejection rates for the four test item types in Experiments 1 and 2. Error bars represent between-subjects standard errors

The rejection rates for the four test item types are shown in Fig. 2 (left panel). We present the statistical analyses below according to the main questions outlined above: (i) did participants learn the regularities? (ii) which factors determine the relative ease of a generalization? and (iii) do participants discriminate between single and double violations?

##### Did participants learn the regularities?

To determine whether participants learned the regularities of the two artificial grammars at all, we conducted three types of comparisons.

First, we compared the rejection rates for the four test item types, separately, to chance performance, operationalized by a chance level of 50% as participants completed a yes/no judgment tasks. That is, above chance performance means that participants should reject grammatical items less often than expected by chance, and violations more often than expected by chance. By contrast, we will refer to below-chance performance when participants reject grammatical items *more* often than expected by chance, or when they reject violations less often than expected by chance. We analyzed the two order conditions separately, as subsequent analyses (in Experiment 2, see below) revealed a statistically significant difference between them.

As shown in Table 1, participants performed significantly better than chance (after Bonferroni correction for multiple comparisons) in both the repetition-/*di*/ and the /*di*/-repetition condition for the grammatical test items, and for those violating both regularities. Performance for single violations did not differ from chance.

**Table 1:** Tests against chance in the different experimental conditions in Experiments 1 and 2

	repetition-/*di*/ order					/*di*/-repetition order				
			significant		above/			significant		above/
			after		below			after		below
Item Type	*t*(19)	*p*_uncorr_	correction	Cohen’s *d*	chance	*t*(19)	*p*_uncorr_	correction	Cohen’s *d*	chance
Experiment 1										
grammatical	11.16	<.0001	∗	5.12	*↑*	6.61	<.0001	∗	3.03	*↑*
repetition violated	1.63	.12	ns	—	—	.21	.84	ns	—	—
/*di*/ violated	2.71	.014	ns	—	—	2.05	.055	ns	—	—
both	4.52	.0002	∗	2.07	*↑*	4.1	.0006	∗	1.88	*↑*
Experiment 2										
grammatical	15.7	<.0001	∗	7.2	*↑*	6.15	<.0001	∗	2.82	*↑*
repetition violated	3.26	.004	∗	1.5	*↓*	1.57	.14	ns	—	—
/*di*/ violated	3.98	.0008	∗	1.83	*↑*	1.89	.075	ns	—	—
both	7.49	<.0001	∗	3.44	*↑*	10.29	<.0001	∗	4.72	*↑*

Effect sizes are Cohen’s *d* for one sample and independent sample t-tests, Cohen’s *d* corrected for dependence between means for pair sample t-tests, and partial *η* ^2^

Second, we compared the rejection rates for the grammatical items with those for the three types of ungrammatical items, as performance on grammatical items can be considered as indicative of maximum learning performance. The results are shown in Table 2. In the repetition-/*di*/ condition, this comparison was significant for the items violating the /*di*/ regularity and both regularities, but not for the items violating the repetition regularity. In the /*di*/-repetition condition, this comparison was significant for all three ungrammatical item types.

**Table 2:** Comparison of the rejection rates for the different violations against the grammatical test items in Experiments 1 and 2

	repetition-/*di*/ order					/*di*/-repetition order				
			significant		above/			significant		above/
			after		below			after		below
Item Type	*t*(19)	*p*_uncorr_	correction	Cohen’s *d*	chance	*t*(19)	*p*_uncorr_	correction	Cohen’s *d*	chance
Experiment 1										
repetition violated	2.75	.013	ns	—	—	4.42	.0003	∗	1.23	*↑*
/*di*/ violated	6.23	<.0001	∗	1.47	*↑*	4.96	<.0001	∗	.9	*↑*
both	8.18	<.0001	∗	1.87	*↑*	6.64	<.0001	∗	1.5	*↑*
Experiment 2										
repetition violated	2.79	.011	ns	—	—	4.84	.0001	∗	.806	*↑*
/*di*/ violated	8.67	<.0001	∗	2.04	*↑*	4.81	.0001	∗	1.5	*↑*
both	12.57	<.0001	∗	2.86	*↑*	9.29	<.0001	∗	2.09	*↑*

Effect sizes are Cohen’s *d* for one sample and independent sample t-tests, Cohen’s *d* corrected for dependence between means for pair sample t-tests, and partial *η* ^2^

Third, we compared rejection rates for test items violating a single regularity to those violating both regularities to test whether the latter were better learned than the former. The results are shown in Table 3. For the items violating the repetition regularity, this comparison was significant in the repetition-/*di*/ condition and marginally significant after Bonferroni correction in the /*di*/-repetition condition, due to lower rejection rates to these single violation items than to the double violation items. The rejection rates for single violations of the /*di*/ regularity did not differ significantly from those for double violations.

**Table 3:** Comparison of the rejection rates for single vs. double violations in Experiments 1 and 2

	repetition-/*di*/ order					/*di*/-repetition order				
			significant		above/			significant		above/
			after		below			after		below
Item Type	*t*(19)	*p*_uncorr_	correction	Cohen’s *d*	chance	*t*(19)	*p*_uncorr_	correction	Cohen’s *d*	chance
Experiment 1										
repetition violated	4.37	.0003	∗	.989	*↑*	2.88	.0095	⋅	.66	(*↑*)
/*di*/ violated	1.37	.186	ns	—	—	1.78	.092	ns	—	—
Experiment 2										
repetition violated	7.51	<.0001	∗	1.72	*↑*	3.79	.0012	∗	.93	*↑*
/*di*/ violated	1.58	.131	ns	—	—	2.52	.012	⋅	.66	(*↑*)

Effect sizes are Cohen’s *d* for one sample and independent sample t-tests, Cohen’s *d* corrected for dependence between means for pair sample t-tests, and partial *η* ^2^

##### Which regularities are easier to learn?

To directly compare how easily the two types of regularities are acquired, we compared the rejection rates for the test items containing single violations (either a /*di*/ or a repetition violation, but not both) in an ANOVA with Regularity (/*di*/ vs. repetition) as a within subject factor and Order (repetition-/*di*/ vs. /*di*/-repetition) as a between-subject factor. The ANOVA yielded a main effect of Regularity, *F*(1,38) =7.76, *p*=.008, $\eta _{p}^{2} = .1696$ due to items violating the /*di*/ regularity incurring higher rejection rates than items violating the repetition regularity. No other main effect or interaction was significant. These results suggest that the /*di*/ regularity was retained better than the repetition-regularity.

##### Do participants discriminate single from double violations?

We compared rejection rates for the means of the two types of test items violating a single regularity with rejection rates for the test items violating both regularities. An ANOVA with within-subject factor Order (repetition-/*di*/ vs. /*di*/-repetition) and Violation Type (single/double) yielded a highly significant main effect of Violation Type, *F*(1,38) =43.2, *p*<.0001, ${\eta _{p}^{2}} = .5319$, as double violations were more often rejected than single violations. No other main effect or interaction was significant.

#### Discussion

The results of Experiment 1 suggest that participants can learn artificial grammars implementing two regularities simultaneously, as they are better than chance at correctly rejecting test items that violate both regularities and at correctly accepting fully grammatical test items. Their performance is at chance for test items violating only one regularity, but they tend to correctly reject items violating the /*di*/ regularity more often than those violating the repetition regularity. These results suggest that the affixation-like /*di*/ regularity is easier to learn than a regularity requiring the comparison of two items. Further, the results indicate a graded response pattern, with good performance on double violations and poorer performance on single violations.

To further probe learning patterns, we tested them in the context of a more subtle type of violation in Experiment 2. In this experiment, ungrammatical test items violated the position rather than the presence of the regularities. For example, strings that violated the /*di*/ regularity did contain the syllable /*di*/, but in the second rather than the first position.

### Experiment 2: Violation of position

#### Methods

##### Participants

Participants were 40 monolingual native English-speaking adults (31 females, 9 males, mean age: 22.50 years, range: 19-42 years), recruited at the University of British Columbia, Vancouver, Canada for course credit.^3^ Participants reported no history of neurological, language or hearing impairment. Half of the participants were randomly assigned to the /*di*/-repetition condition and half to the repetition-/*di*/ condition.

##### Stimuli

The two artificial grammars that generated the sequences presented in the familiarization phase were identical to those used in Experiment 1.

For the test phase, novel grammatical and ungrammatical sequences were created. In contrast to Experiment 3, where the ungrammatical strings did not implement one regularity or both, the ungrammatical sequences in Experiment 3 implemented the regularities, but in an incorrect, non-edge position. We call this a violation of position. Specifically, the ungrammatical sequences could violate the repetition regularity, the /*di*/ regularity or both. This resulted in four types of test items: (i) grammatical items, identical to those used in Experiment 1 (/*di*/ABCDD or AABCD/*di*/, depending on the grammar participants had been familiarized with), (ii) repetition violations (/*di*/ABCCD or ABBCD/*di*/), (iii) /*di*/ violations (A/*di*/BCDD or AABC/*di*/D), and (iv) violations of both the repetition and the /*di*/ regularity (A/*di*/BCCD, ABBC/*di*/D). For each test item type, 9 items were created, for a total of 36 test items for condition. The sequences were synthesized in the same way as in Experiment 1.

##### Procedure

The procedure was identical to Experiment 3.

#### Results

The rejection rates for the four test item types are shown in Fig. 2 (right panel). We present the statistical analyses in the same way as for Experiment 3.

##### Did participants learn the regularities?

To determine whether participants learned the regularities of the two artificial grammars at all, we conducted three types of comparisons. First, we compared the rejection rates for the four test item types separately to chance performance. As shown in Table 1, participants performed significantly better than chance (after Bonferroni correction for multiple comparisons) in the repetition-/*di*/ condition for the grammatical test items, the /*di*/ violations, and double violations. However, they performed significantly below chance for the repetition violation; in other words, they had a tendency to treat them as legal items. In the /*di*/-repetition condition, they performed significantly better than chance for the grammatical test items, and for the items violating both regularities, but their performance was indistinguishable from chance for the single violations.

Second, we compared the rejection rates for the grammatical items with those for the three types of ungrammatical items. The results are shown in Table 2. In the repetition-/*di*/ condition, this comparison was significant for the items violating the /*di*/ regularity and both regularities, but not for the items violating the repetition regularity. In the /*di*/-repetition condition, this comparison was significant for all three ungrammatical item types. These results thus parallel those of Experiment 1.

Third, we compared rejection rates for test items violating a single regularity to those violating both regularities to test whether the latter were better learned than the former. The results are shown in Table 3. For items violating the repetition regularity, this comparison was significant in both the repetition-/*di*/ condition and the /*di*/-repetition condition. For the items violating the /*di*/ regularity, this comparison was marginally significant after Bonferroni correction in the /*di*/-repetition condition, and non-significant in the repetition-/*di*/ condition.

##### Which regularities are easier to learn?

To directly assess which of the two types of regularities was retained better, we compared the rejection rates for the test items containing single violations (either a /*di*/ or a repetition violation, but not both) in an ANOVA with Regularity (/*di*/ vs. repetition) as a within subject factor and Order (repetition-/*di*/ vs. /*di*/-repetition) as a between-subject factor. The ANOVA yielded a main effect of Regularity, *F*(1,38) =9.21, *p*=.0043, ${\eta _{p}^{2}} = .1951$ due to items violating the /*di*/ regularity incurring higher rejection rates than items violating the repetition regularity. The main effect of Order showed a trend towards significance, *F*(1,38)=3.61, *p*=.065, ${\eta _{p}^{2}} = .0868$. The Regularity × Order interaction was also significant, *F*(1,38)=6.94, *p*=.012, ${\eta _{p}^{2}} = .1544$. As LSD post hoc tests showed, this interaction was carried by higher rejection rates for the /*di*/ violation than for the repetition violation in the repetition-/*di*/ order, *p*=.0003, and by higher rejection rates for the repetition violation than for the /*di*/ violations in the /*di*/-repetition order, *p*=.008. In other words, violations of the sequence-final regularity were easier to detect.

##### Do participants discriminate single from double violations?

We compared rejection rates for the means of the two types of test items violating a single regularity with rejection rates for the test items violating both regularities. An ANOVA with within-subject factor Order (repetition-/*di*/ vs. /*di*/-repetition) and Violation Type (single vs. double) yielded a significant main effect of Violation Type, *F*(1,38)=109.1, *p*<.0001, ${\eta _{p}^{2}} = .7417$, as double violations were more often rejected than single violations. No other main effect or interaction was significant.

#### Discussion

Like in Experiment 1, participants in Experiment 2 showed an overall ability to learn the artificial grammars they were exposed to. Unlike in Experiment 1, however, their performance was modulated by order effects. In the repetition-/*di*/ order, they showed rejection rates that were lower than chance for the repetition violations, indicating incorrect performance, but rejection rates that were better than chance for the /*di*/ violations. It thus appears that, when more subtle violations are involved, order effects related to memory constraints on serial order play an important role: the repetition-based regularity, which already proved less salient in the violation of presence condition in Experiment 1, became even more challenging for participants when it appeared in a sequence-initial position. This result is not predicted by either account, but it is not unexpected under a perceptual and memory primitive based account. We will discuss it further below.

The difference between single vs. double violations shows the same pattern as in Experiment 1, with double violations being more readily rejected than single violations.

### Are sequence-final regularities easier to learn than sequence-initial regularities?

In Experiment 2, we found that violations of the repetition-pattern were more easily detected in sequence-final positions than in sequence initial positions. Furthermore, visual inspection of Fig. 2 shows that there is at least a numeric advantage for single violations of a regularity when it occurs at the sequence-end as compared to when it appears at the onset.

To further analyze this impression, we jointly analyzed the rejection rates for single violations from Experiments 1 and 2 in a generalized linear mixed model, fitted to trial-by-trial data, using a binomial link function. The initial model comprised fixed factor predictors for Violated Regularity (/*di*/ vs. repetition), Order (repetition-/*di*/ vs. /*di*/-repetition) and Violation Type (presence vs. position, i.e., Experiment 1 vs. Experiment 2) as well as all of their interactions. We included random intercepts for participants and trials. We kept only those interactions and random intercepts that contributed to the model likelihood. In the final model, we included the three main effects, the interaction between Order and Violation Type as well as a random intercept for participants.

The results of the model are shown in Table 4. This model revealed that violations of the /*di*/-regularity led to significantly higher rejection rates than violations of the repetition-regularity, *β* = .45, *S* *E*=.13, *Z*=3.34, *p*=.0008, confirming that the /*di*/-regularity was more salient. We also found that rejections rates in the repetition-/*di*/ condition were significantly lower than in the /*di*/-repetition condition, *β*=−1.15, *S* *E*=.24, *Z*=4.73, *p*<.00001, and, importantly that rejection rates in the repetition-/*di*/ condition were increased for violations of the /*di*/-regularity, *β*=1.64, *S* *E*=.23, *Z*=7.16, *p*<.00001. This latter result reflects the recency effect discussed above. To see this recency effect more clearly, we excluded the main effect of order from the above model.

**Table 4:** Results of a generalized linear mixed model with binomial link function, restricted to trials with single violations

	*β*	*SE*	*Z*	*p*
Intercept	0.23	0.18	1.25	0.21
Order = repetition-*di*	−1.15	0.24	−4.73	< .00001
Violation Type = Position	0.14	0.21	0.68	0.494
Violated Regularity = *di*	0.45	0.13	3.34	.0008
(Order = repetition-*di*):(Violated Regularity = *di*)	1.64	0.23	7.16	< .00001

The final model specification was Rejection Order + ViolationType + ViolatedRegularity + Order:ViolatedRegularity + (1 | Participant)

The results of this restricted model are shown in Table 5. The model revealed again that violations of the /*di*/-regularity led to significantly higher rejection rates than violations of the repetition-regularity, *β*=.45, *S* *E*=.13, *Z*=3.34, *p*=.0008. Crucially, the interaction between Order and Violation Type revealed that, when the repetition regularity was violated, rejection rates were reduced in the repetition-/*di*/ condition compared to the in the /*di*/-repetition condition, *β*=−1.15, *S* *E*=.24, *Z*=4.73, *p*<.00001, while, when the *di*-regularity was violated, rejection rates received a small boost in the repetition-/*di*/ condition, *β*=−.49, *S* *E*=.25, *Z*=1.98, *p*<.047.^4^

**Table 5:** Results of the overall analysis of Experiments 1 and 2, using a model with the specification Rejection Order + ViolationType + ViolationOfRepetition + ViolationOfDi + Order:ViolationOfRepetition + Order:ViolationOfDi + ViolationType:ViolationOfDi + ViolationOfRepetition:ViolationOfDi + (1 | Participant)

	*β*	SE	*Z*	*p*
Intercept	−1.67	0.19	−9.00	< .00001
Order = repetition-*di*	−0.54	0.24	−2.26	.024
Violation Type = Position	−0.11	0.20	−0.56	0.578
Violation of Repetition = yes	2.03	0.14	14.69	< .00001
Violation of *di* = yes	2.19	0.16	13.45	< .00001
(Order = repetition-*di*):(Violation of Repetition = yes)	−0.62	0.18	−3.48	.0005
(Order = repetition-*di*):(Violation of *di* = yes)	0.96	0.19	5.16	< .00001
(Violation Type = Position):(Violation of *di* = yes)	0.55	0.17	3.23	.001
(Violation of repetition = yes):(Violation of *di* = yes)	−0.90	0.17	−5.23	< .00001

### Overall analysis

In the next analysis, we analyze all conditions of the combined results of Experiments 1 and 2 (and not only the data for single violations, as in the previous analysis), fitting a generalized linear mixed model with a binomial link functions to trial-by-trial rejection data. The initial model specification included the fixed factors Order (repetition-/*di*/ vs. /*di*/-repetition) and Violation Type (presence vs. position), Repetition Violation (yes vs. no), *di* Violation (yes vs. no), all interactions as well as random intercepts for participants and trials. We retained only those interactions and random intercepts that contributed to the model likelihood. The final model included the four main effects and interactions between Order and Repetition Violation, between Order and *di* Violation, between Violation Type and *di* Violation and between Repetition Violation and *di* Violation. We included only a random intercept for participants.

This model revealed that rejection rates were higher when the *di* regularity was violated, *β*=2.19, *S* *E*=.16, *Z*=13.45, *p*<.00001, and when the repetition regularity was violated, *β*=2.03, *S* *E*=.14, *Z*=14.69, *p*<.00001. An interaction between these factors suggested that rejection rates were somewhat lower when both regularities were violated than would be expected from simply adding the contributions of the two rejection rates, *β*=−.90, *S* *E*=.17, *Z*=5.23, *p*<.00001.

Rejection rates were somewhat lower in the repetition-/*di*/ condition, *β*=−.54, *S* *E*=.24, *Z*=−2.26, *p*=.024. An interaction with Repetition Violation suggested that this effect was somewhat more pronounced when the repetition regularity was violated, *β*=−.62, *S* *E*=.18, *Z*=−3.48, *p*=.0005, and substantially less pronounced when the *di* regularity was violated, *β*=.96, *S* *E*=.19, *Z*=5.16, *p*<.00001. This result reflects the recency effect discussed above.

Finally, an interaction between Violation Type and *di* Violation suggested that rejection rates were somewhat higher when positional violations were used and the *di* violation was violated, *β* = .55, *S* *E*=.17, *Z*=3.23, *p*=.001.

### To what extent are extant Bayesian models consistent with the data?

We now take advantage of the explicit nature of Frank and Tenenbaum’s (2013) model to ask to what extent it is compatible with the results of the current experiments. In Appendix A, we derive the equations for the posterior probabilities of the test items. Specifically, in line with Frank and Tenenbaum’s (2013) models, we assume that the model considers four kinds of rules: (i) a default rule that is true of all strings (and thus of *S* ^6^ possible 6-syllable strings generated from *S* strings); (ii) a repetition rule that detects repeated syllables in a specific position in a string (and that is compatible with *S* ^5^ strings); (iii) an affixation rule that detects specific syllables in specific positions (and that is compatible with *S* ^5^ strings); and (iv) the conjunction rule of the latter two rules (that is compatible with *S* ^4^ strings).

In order to evaluate their model, Frank and Tenenbaum (2013) used “surprisal” as a measure of the model output for yes/no grammaticality judgments (e.g., of Endress et al.’s (2007) experiments), which indicates how “surprising” a test item is after having heard the familiarization items. (Formally, surprisal is the negative logarithm of the posterior probability of a test item, and reflects how much information is carried by the test item in the context of the prior familiarization). We will thus adopt this metric as one of the measures to evaluate our own simulations. However, this is not an appropriate measure to compare to empirical acceptance or rejection rates of strings, as it is not a probability (M. Frank, personal communication). In addition to surprisal, we thus evaluate the model with the posterior probability of the test items, given the training items.

However, raw posterior probabilities are extremely low, predicting that all items should rejected. To circumvent this problem, we also evaluate the model *as if* the experiments used three-alternative forced choice tasks between test items, where participants (or the model) are familiarized with the training strings, and then have to choose between grammatical items, single violations and double violations. Modeling a forced choice task thus allows us to use the relative likelihoods of the test items, and thus to work around the low posterior probabilities.

We analyze two versions of the model, the original one with the conjunction rule, as well as a version without the conjunction rule, in order to better assess the contribution of this rule to fitting experimental data.

#### Original model

**Fig. 3:**
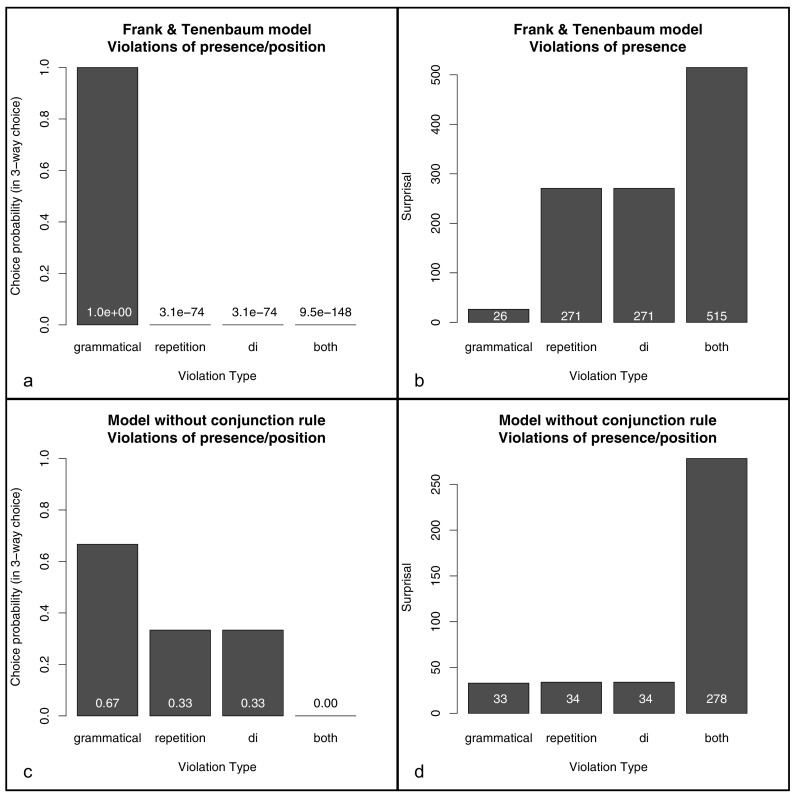
Results of a Bayesian model based on Frank and Tenenbaum (2013). The model results are identical for violations of presence and of position. To compare the modeling results to our experimental results, we assume that there is a monotonic relation between posterior probabilities and endorsement rates, and between surprisal and rejection rates

The posterior probabilities and surprisals for grammatical items, violations of a single feature (repetition or affixation pattern) and violations of both features are calculated in Appendix 1, where |*T*| is the number of training items and *S* is the number of syllables. We then treated the different test items *as if* participants had to choose among them as alternatives in a three-way forced choice task. That is, we assumed that the model was familiarized with the training items, and then had to choose in each trial between grammatical items, single violations and double violations.^5^ As shown in Fig. 3a, we found that the probability of choosing grammatical items is 1, while the probability of choosing any other items is zero.

In other words, the model should exclusively choose grammatical items, and reject all other items. Further, it does not discriminate between items violating the /*di*/ regularity and items violating the repetition regularity, and items violating both regularities. This behavior contrasts markedly with that of our participants. To see why this is the case, consider our mixed model analyses above, and recall that the slopes and intercepts when predicting endorsement rates are same as when predicting rejection rates except for the sign (since a logistic transform has been applied). Frank and Tenenbaum’s (2013) model predicts that either violation of either regularity is sufficient for an item to be rejected. As a result, the interaction between the predictors corresponding to the violations of the two regularities must cancel out the effect of one of the violations. After all, if one violation is sufficient to lead to rejection of an item, a second violation would lead to a rejection rate of more than 100% if it is not cancelled out by the interaction term. In contrast, in our mixed model analyses, the coefficient of the interaction was less than half of that of either violation, suggesting that the behavior of actual participants is much more gradual than Frank and Tenenbaum’s (2013) model suggests.

For completeness, surprisal values are shown in Fig. 3b. The central results are as before: the model does not discriminate between violations of the /*di*/ regularity and violations of the repetition pattern, nor between violations of the presence of a regularity (e.g., strings of the form ABCDEF after familiarization with AABCD/*di*/, where neither regularity exists in the test string) and violations of its position (e.g., strings of the form ABBC/*di*/D after familiarization with AABCD/*di*/, where the test string contains both a position and the /*di*/ syllable, but in incorrect positions). However, at least when equating surprisal to rejection rates, the model predicts that participants should be about 7.5 times as likely to reject double violations than single violations.

#### Model without conjunction rules

Given that Frank and Tenenbaum’s (2013) explanation of Gerken’s (2010) data relies on the specificity of the conjunction rule, we also calculate the posterior probabilities of a model that does not comprise such conjunction rules to allow for a more general evaluation of the model.

Fig. 3c shows the choice probabilities in a three way choice. The probability of choosing grammatical items over single violation or double violation items is about 2/3. (In this three-way choice, we just represent single-violation items as a single choice. However, in a choice between items violating both regularities, items violating the /*di*/ regularity, items violating the repetition regularity, and grammatical items, the choice probability for grammatical items would be 1/2, and more generally 2/(2 + *N*), where *N* is the number of single violation items entering the choice. In Frank and Tenenbaum’s (2013) original model, the number of single violation items does not noticeably affect choice probabilities).

Finally, Fig. 3d shows the surprisal values. The central results are as before: the model does not discriminate between violations of the /*di*/ regularity and violations of the repetition pattern, nor between violations of the presence of a regularity (e.g., strings of the form ABCDEF after familiarization with AABCD/*di*/, where neither regularity exists in the test string) and violations of its position (e.g., strings of the form ABBC/*di*/D after familiarization with AABCD/*di*/, where the test string contains both a position and the /*di*/ syllable, but in incorrect positions). Further, the model predicts that participants should be about 7.6 times as likely to reject double violations than single violations.

In sum, extant Bayesian models of rule learning seem inconsistent with the data presented here. In particular, they do not predict the graded nature of the response, the difference between the learnability of the repetition and the *di* regularity or the observed order effects. These results thus add to more general issues that need to be clarified with respect to such models (see Endress, 2013, for discussion). For example, how do learners “know” which regularity is narrower? According to Frank and Tenenbaum’s (2013) models, infants keep track of all the syllables they hear during familiarization, use them to construct all possible triplets, and check for each triplet whether it is consistent with any conceivable rule. For example, if infants encountered a total of three syllables, they would generate all 27 triplets that can be formed with these syllables, and realize that, of these 27 triplets, 6 follow an ABB pattern (e.g., *pu-li-li*), 3 follow an AAA pattern (where all three syllables are identical), and so on. This allows them to count the number of triplets that is consistent with each generalization and, therefore, to choose the narrowest one. While Frank and Tenenbaum (2013) acknowledged that this model is implausible, it is unclear how infants might possibly know the number of triplets consistent with each generalization if they do not generate all possible triplets.

Moreover, it is not clear whether infants actually represent conjunction rules of the type mentioned above. Possibly, they might just have learned that items end in /*di*/ and start with a repetition, but without joining these patterns into a conjunction rule.

In sum, extant Bayesian models of rule learning need to improve their empirical fit to the data as well as the psychological meaning/plausibility of their assumptions.

## General discussion

In the present study, we investigated how human adults learn when they are exposed to strings that conform to multiple patterns simultaneously. Participants were presented with one of two kinds of strings. They were exposed to strings that started with a repeated syllable and ended with /*di*/ (repetition-/*di*/ order), or they were exposed to strings that started with /*di*/ and ended with a repeated syllable (/*di*/-repetition). We obtained three major results. First, participants learned both regularities simultaneously. They had a strong tendency to accept novel items that were grammatical, strongly reject novel items that violated both regularities, and reject at intermediate rates the items that violated only one of the regularities. Second, violations of the repetition-pattern were less salient to participants than violations of the regularity constraining the start or end syllables. Third, violations of regularities at the end of sequences were more salient than violations at the beginning of sequences.

These results reflect fundamental constraints on the nature of the processes involved in the acquisition of rule-like regularities, and give crucial insight into the patterns of occurrence of certain morphosyntactic regularities across the world’s languages. We will now discuss these issues in turn.

### How are rule-like generalizations learned

As reviewed in the introduction, there are two major views on how rules similar to those used here are learned. On the one hand, learners might rationally optimize some objective function, and learn the most specific rule that is compatible with the data (e.g., Frank & Tenenbaum, 2013; Gerken, 2010). On the other hand, such regularities might be detected by simpler perceptual or memory primitives.

The present results clearly support the primitives view, for at least three reasons. First, the specificity of a rule does not seem to influence how easily a rule is acquired (see also Endress, 2013). As mentioned above, there is an equal number of strings that can be generated with either the repetition-pattern or the pattern constraining the initial or final syllable. Nonetheless, participants seem to learn the syllable-based pattern better than the repetition pattern.

Second, the relative rejection rates for the test items fit neither Frank and Tenenbaum’s (2013) original model of rule learning, nor our version not comprising the conjunction rule. Specifically, our results show intermediate rejection rates for items that violate a single rule compared to grammatical test items and test items that violate both rules. The predictions of Bayesian models of rule learning seem at odds with these results. First, such models do not predict any difference between violations of repetition-patterns and affixation patterns, between violations of position and of existence, between serial positions, and so forth. Of course, it is possible to construct a Bayesian model that does account for such data, for example by changing the prior probabilities of the rules. However, when auxiliary assumptions are added without independent motivation, then models become ad hoc, and it becomes hard to distinguish between predictions that result from ad hoc assumptions, and predictions that result from the underlying Bayesian machinery. We believe that the perceptual or memory primitives account is more attractive in this respect as rule salience and learnability can be tested and measured empirically.

Second, when evaluated using Luce’s choice rule (reflecting a three alternative forced-choice task), Frank and Tenenbaum’s (2013) predicts that participants should *never* choose any items that are not fully grammatical. This model behavior is due to a combination of two factors: They assume that learners represent a conjunction rule (i.e., the conjunction of a repetition-rule and a affixation-rule), and they assume that participants evaluate rules using the size principle. These assumptions conspire to make the posterior probabilities of test items respecting the conjunction rule many orders of magnitude larger than that of test items not respecting it, in our experiments by a factor of 3×10^73^. In line with this interpretation, a variant of Frank and Tenenbaum’s (2013) model not comprising the conjunction rule predicts that, when choosing between grammatical items, items with a single violation, and items with a double violation, participants should choose the grammatical item about 2/3 of the time, and the item with the single violation the rest of the time (though the exact choice probabilities depend on how it is calculated), which is consistent with the empirical result that the choice between grammatical items and single violations is graded.^6^ In this model, the preference for grammatical items over single-violations is due to the fact that grammatical items conform to two rules (that happen to be equally specific) rather than a single one, which, we believe, is a conclusion that is consistent with virtually any modeling framework. The current results suggest that participants do not represent conjunction rules, and support Endress’s (2013) suggestion that the infants’ difficulty to recognize single-violations as opposed to grammatical items in Gerken’s (2010) experiments was due to the fact that grammatical items conform to two rules rather than a single one, and not to the specificity of a putative conjunction rule.

Interestingly, this conclusion also seems to be in line with what is known about perception in general. In vision, it is easier to search for targets defined by single features (e.g., a blue letter among green and brown letters, or an S among T’s and X’s) than to search for feature conjunctions (e.g., a green T among brown T’s and green X’s; e.g., Treisman & Gelade, 1980; Wolfe, 2003). In contrast, while Frank and Tenenbaum’s (2013) model also performs a search, albeit among possible rules, the model assumes that conjunction rules should be learned preferentially.

All these results mesh well with the primitives view. The very point of this view is that humans (and other animals) have propensities to learn certain patterns, and that some patterns are empirically more salient than others. Moreover, it is not unexpected that the repetition-pattern is somewhat harder to learn than the /*di*/ regularity, possibly because it involves two items rather than one. Likewise, the gradual difference in rejection rates between single vs. double violations is not unexpected either, as participants might notice that there is something “right” about items that violate a single regularity if they learn both rules independently. However, *a priori* considerations are often misleading and determining the saliency of a pattern or the relative saliency of two patterns remains an empirical question.

Importantly, the present results also reveal a finding that is not predicted by either account: regularities located at sequence-offsets seem to be more salient than regularities located at sequence-onsets. In other words, there is a recency effect for regularities. While we are not aware of empirical studies investigating how experimental parameters affect the relative strength of primacy and recency effects in the case of memory for serial order, the literature on item memory suggests that their relative strength might depend on different factors, for example the ratio of the retention interval and the interstimulus interval (e.g., Knoedler, Hellwig, & Neath, 1999; Neath, 1993). As a result, it would have been difficult to make straightforward predictions about this point. However, as we will discuss in more detail below, this finding explains important cross-linguistic regularities.

### Implications for language

We suggest that the rules presented here are learned through simple perceptual or memory primitives. These primitives appear to be shared by non-human animals (e.g., Chen et al., 2016; Endress, Carden, Versace, & Hauser, 2010; Endress, Cahill, et al., 2009; Giurfa et al., 2001; Hauser & Glynn, 2009; Martinho & Kacelnik, 2016; Murphy, Mondragon, & Murphy, 2008; Neiworth, 2013; van Heijningen et al., 2013) who clearly do not acquire language. It turns out, however, that these basic mechanisms seem to explain a number of cross-linguistic generalizations, which, in turn, suggest that language acquisition and processing might rely on some similar mechanisms (Endress, Nespor, & Mehler, 2009).

For example, across the languages of the world, prefixation and suffixation patterns are much more frequent than infixation patterns (such as *fan-fucking-tastic*; e.g., McCarthy, 1982); this observation meshes well with the fact that in artificial grammar learning experiments, participants predominantly learn regularities that involve the edges of constituents as opposed to other positions within sequences. Further, when infixation patterns occur, they tend to be located near edges of constituents or next to a stressed unit (Yu 2007).

Furthermore, across the languages of the world, the relative frequency of prefixation and suffixation is reflected by the experiments presented here. In fact, across the 828 languages that have been identified in the WALS (http://wals.info/) as having some amount of inflectional morphology, 529 show some predominance of suffixation vs. 152 showing a predominance of prefixation (with 147 languages having equal amounts of pre- and suffixation). Thus, suffixation is about 3-4 times more common than prefixation (Dryer 2013), which fits well with the recency effect obtained above (see also Endress & Hauser, 2011 for more evidence that suffixes are easier to learn than prefixes). Further, reduplication, which our study has found to be more challenging to learn than single syllable affixation, is indeed less frequent and/or used for fewer morphological functions in the world’s languages than the affixation of a single marker.

Two caveats are in order. First, although our experiments address language learning in general, and intend to shed light on language acquisition, we nevertheless tested adult, and not infant participants for the practical reasons mentioned earlier. Infants and adults differ in some of their language learning abilities, possibly due to their different cognitive and memory capacities (e.g., Newport, 1990; Newport & Neville, 2001), or because they have outgrown their critical period for language acquisition (e.g., Lenneberg, 1967). For example, having larger memory and attentional capacities, adults are better able to store individual items, exceptions and irregular forms, and may thus be better statistical learners, while infants, given their limited memory capacity, might focus on extracting rules and generalizations in order to capture as much as possible of a given dataset (e.g., Finn & Hudson Kam, 2008; Gervain et al., 2013; Hudson Kam & Newport, 2005; Marchetto & Bonatti, 2013, 2015; Newport, 1990). However, adults and infants are expected to differ less in their perceptual and memory primitives. Indeed, sensitivity to repetition has been found in infants as young as newborns (Antell, Caron, & Myers, 1985; Gervain, Macagno, et al., 2008; Gervain et al., 2012). Furthermore, implicit artificial grammars have been argued to recruit similar neural correlates as natural languages (e.g., Friederici, Steinhauer, & Pfeifer, 2002; Bahlmann, Schubotz, & Friederici, 2008).

Second, our adult participants were speakers of English, and might have brought their language-specific knowledge to the laboratory. While it is still interesting to note that those patterns that are easier to learn are also those that are more frequent cross-linguistically, it is important to test with young infants and non-human animals whether these effects can be found irrespective of language experience.

In addition to typological evidence, studies in language acquisition also suggest that the ends of words are more salient, and that suffixation may be universally more common precisely because it facilitates learning. (Slobin 1973), for instance, makes the empirical generalization, on the basis of early production data from 40 typologically different languages, that post-verbal and post-nominal markers are acquired earlier than pre-verbal and prenominal ones, and attributes this to the greater salience of word ends as compared to word beginnings (operating principle A: “pay attention to the ends of words”, Slobin, 1973). Indeed, the analysis of a corpus of child-directed English suggests that suffixes predict the stems grammatical category with greater reliability than prefixes, and that participants can better learn the grammatical category of word stems in an artificial grammar study on the basis of suffixes than on the basis of prefixes (St Clair, Monaghan, & Ramscar, 2009). As a flip side of the idea that word ends are preferentially attended to when learning morphological regularities, psycholinguistic studies whereby adults needed to learn new word-object associations suggest that participants associate word beginning more strongly with the words’ referents than they do word ends (see Creel & Dahan, 2010 and references therein). As a result, there does not seem to be an overall processing advantage for word ends. Rather, the end advantage we observe seems mostly related to morphological-like processing.

## Conclusion

While language is learned only by humans, certain basic abilities present in other animals might be the proximate mechanisms by which crucial aspects of language are acquired, and might also constrain the expressed form of language (see also Wang & Seidl, 2015). Given the amenability of such mechanisms to experimental manipulations, they might be a unique opportunity to understand the mechanistic and evolutionary basis of certain crucial aspects of language acquisition and use.

## Appendix A: General model equations for the original model

In line with Frank and Tenenbaum’s (2013) conventions, *e*_*k*_ is the *k* ^th^ test item, *r*_*j*_ is the *j* ^th^ rule, *R* is the set of all rules, and *T* is the set of all training strings. |.| denotes the number of items in a set. Below, we will call *R* ^*c*^ the set of rules that is compatible with *all* training strings, and ${R_{j}^{c}}$ the set of rules that is compatible with all training strings as well as the (test) string *j*. Further, we have the following equation (see Frank and Tenenbaum’s (2013) Eq. 8, where the sum and the product should have been switched):

1 $$\begin{array}{@{}rcl@{}} p(e_{k}|T) & = & {\sum}_{r_{j} \in R} p(e_{k}|r_{j})p(r_{j}|T) \end{array} $$

Frank and Tenenbaum (2013) assume here conditional independence of the training strings and the test strings, given a rule. Further, from their Eqs. 1 to 3, we have:

2 $$\begin{array}{@{}rcl@{}} p(e_{k}|r_{j}) & = & \left\{\begin{array}{ll} \frac{1}{|r_{j}|} & \text{if }e_{k}\text{ is compatible with }r_{j}\\ 0 & \text{otherwise} \end{array} \right. \end{array} $$

3 $$\begin{array}{@{}rcl@{}} p(r_{j}|T) & = & \frac{p(T|r_{j}) p(r_{j})}{{\sum}_{r^{\prime} \in R} p(T|r^{\prime}) p(r^{\prime})} \end{array} $$

4 $$\begin{array}{@{}rcl@{}} & =& \frac{p(T|r_{j})}{\sum\limits_{r^{\prime} \in R} p(T|r^{\prime})}\\ & = & \frac{{\prod}_{t_{i} \in T} p(t_{i}|r_{j})} {{\sum}_{r^{\prime} \in R}\left\{{\prod}_{t_{i} \in T} p(t_{i}|r^{\prime}) \right\}}\\ p(r_{j}|T) &= & \frac{\left( \frac{1}{|r_{j}|}\right)^{|T|}} {{\sum}_{r^{\prime} \in R^{c}} \left( \frac{1}{|r^{\prime}|}\right)^{|T|}} \end{array} $$

These equations follow from Frank and Tenenbaum’s (2013) use of a uniform prior over rules, of the conditional independence of test strings given a rule, and of the assumption of strong sampling.

From these equations, we can derive an expression for the posterior probability of a test item, given the training strings:

5 $$\begin{array}{@{}rcl@{}} p(e_{k}|T) & \!= & {\sum}_{r_{j} \in {R_{k}^{c}}} \frac{1}{|r_{j}|} \frac{\left( \frac{1}{|r_{j}|}\right)^{|T|}} {{\sum}_{r^{\prime} \in R^{c}} \left( \frac{1}{|r^{\prime}|}\right)^{|T|}} \!= \frac{\sum\nolimits_{r^{\prime} \in {R_{k}^{c}}} \left( \frac{1}{|r^{\prime}|}\right)^{|T|+1}} {\sum\nolimits_{r^{\prime} \in R^{c}} \left( \frac{1}{|r^{\prime}|}\right)^{|T|}} \\ \end{array} $$

In the numerator, we sum over all rules that are compatible with all training items and with the test item *e*_*k*_. In the denominator, we sum over all rules that are compatible with all training items.

Let $\hat {r}$ be the most specific rule. Then $1/|r_{j}| \leq 1/|\hat {r}|$ for all *j*. It follows that

6 $$\begin{array}{@{}rcl@{}} p(e_{k}|T) & \leq & \frac{1}{|\hat{r}|} \end{array} $$

In our experiments, the most specific rule generates *S* ^4^ strings, where *S* is the number of syllables. With 97 syllables as in our experiments, the posterior probability of any test item is thus at most .000001 %. As a result, all test items should be rejected.

## Appendix B: Formulae for the original model

### Posterior probability of test items

We now calculate the posterior probabilities for the rules used here. Let *S* be the number of syllables. Given that we use strings with six syllables, there are *S* ^6^ strings in total, *S* ^5^ strings that have a repetition in a given edge or a specific affix syllable, and *S* ^4^ items conforming to the conjunction rule of affix syllable and reduplication. This allows us to calculate the posterior probabilities for ungrammatical items, items that conform to one of the rules, and items that conform to both rules. We will call these items *e*_*k*,0_, *e*_*k*,1_ and *e*_*k*,2_, respectively, where the second index refers to the number of rules to which an item conforms.

In the denominator of Eq. 5, we need to sum over the default rule, the affixation rule, the repetition rule as well as the combination rule. This yields (1/*S* ^6^)^|*T*|^+2(1/*S* ^5^)^|*T*|^+(1/*S* ^4^)^|*T*|^=(1/*S* ^4^)^|*T*|^((1/*S* ^|*T*|^)^2^+2(1/*S* ^|*T*|^)+1)=(1/*S* ^4^)^|*T*|^(1+1/*S* ^|*T*|^)^2^.

In the numerator of Eq. 5, we have to sum over the applicable rules for each item. For ungrammatical items, this is just the default rule, for items conforming to one rule, we add the corresponding rule, and for grammatical items, we need to add a second rule as well as the conjunction rule. In equations, this yields:

7 $$\begin{array}{@{}rcl@{}} p(e_{k,0}|T) & = & \frac{\left( \frac{1}{S^{6}}\right)^{|T|+1}} {\left( \frac{1}{S^{4}}\right)^{|T|} \left( 1 + \frac{1}{S^{|T|}}\right)^{2}} \end{array} $$

8 $$\begin{array}{@{}rcl@{}} & = & \frac{1}{S^{2|T| + 6}} \frac{1}{\left( 1 + \frac{1}{S^{|T|}}\right)^{2}} \end{array} $$

9 $$\begin{array}{@{}rcl@{}} & \approx & \frac{1}{S^{2|T| + 6}} \end{array} $$

10 $$\begin{array}{@{}rcl@{}} p(e_{k,1}|T) & = & \frac{\left( \frac{1}{S^{6}}\right)^{|T|+1} + \left( \frac{1}{S^{5}}\right)^{|T|+1} } {\left( \frac{1}{S^{4}}\right)^{|T|} \left( 1 + \frac{1}{S^{|T|}}\right)^{2}} \end{array} $$

11 $$\begin{array}{@{}rcl@{}} & = & \frac{1}{S^{|T| + 5}} \frac{1 + \frac{1}{S^{|T|+1}}} {\left( 1 + \frac{1}{S^{|T|}}\right)^{2}}\\ \end{array} $$

12 $$\begin{array}{@{}rcl@{}} & \approx & \frac{1}{S^{|T|+5}} \end{array} $$

13 $$\begin{array}{@{}rcl@{}} p(e_{k,2}|T) & = & \frac{\left( \frac{1}{S^{6}}\right)^{|T|+1} + 2 \left( \frac{1}{S^{5}}\right)^{|T|+1} + \left( \frac{1}{S^{4}}\right)^{|T|+1}} {\left( \frac{1}{S^{4}}\right)^{|T|} \left( 1 + \frac{1}{S^{|T|}}\right)^{2}} \end{array} $$

14 $$\begin{array}{@{}rcl@{}} & = & \frac{1}{S^{4}} \frac{\frac{1}{S^{2|T|+2}} + 2\frac{1}{S^{|T|+1}} + 1} {\left( 1 + \frac{1}{S^{|T|}}\right)^{2}} \end{array} $$

15 $$\begin{array}{@{}rcl@{}} & = & \frac{1}{S^{4}} \frac{\left( 1 + \frac{1}{S^{|T|+1}}\right)^{2}} {\left( 1 + \frac{1}{S^{|T|}}\right)^{2}} \end{array} $$

16 $$\begin{array}{@{}rcl@{}} & \approx & \frac{1}{S^{4}} \end{array} $$

The approximations result from the fact that the dropped terms are much smaller than 1; for example, with *S* =97 and |*T*| =36, 1/*S* ^|*T*|^=3×10^−72^.

Further, it is easy to see that, as one increases the number of syllables, all *p*(*e*_*k*_|*T*)’s converge to zero. That is, Frank and Tenenbaum’s (2013) model makes the prediction that if one presents participants with the very same training examples, but makes them aware before the experiment that there are more possible syllables, they should essentially reject all test strings. It seems reasonable to conclude that this is not how actual humans behave.

## Surprisal for the test items

Given the above formulae, we can also calculate the surprisal *s* for each test item. This is given by (where log represents the logarithm with basis 2):

17 $$\begin{array}{@{}rcl@{}} s(e_{k,0}) & = & (2|T| + 6) \log (S) \end{array} $$

18 $$\begin{array}{@{}rcl@{}} s(e_{k,1}) & = & (|T| + 5) \log (S) \end{array} $$

19 $$\begin{array}{@{}rcl@{}} s(e_{k,2}) & = & 4 \log (S) \end{array} $$

### Choice probabilities

Below, we calculate the choice probabilities for test items *as if* the experiments were conducted as a choice experiment (while the experiments really use yes/no recognition judgements). We use Luce’s choice rule, that is, if participants have to choose among *N* possibilities associated with a probability *p*_*i*_, the *j* ^*t**h*^ item is chosen with the following probability:

20 $$\begin{array}{@{}rcl@{}} \frac{p_{j}}{{\sum}_{i=1}^{N} p_{i}} \end{array} $$

We calculate two kinds of situation, one where grammatical items and items with one violation are pitted against a baseline of ungrammatical items, and one where participants have to make a three-way choice among all three types of items.

### Choices against ungrammatical items as a baseline

Below, we show the choice probabilities in two-alternative choices where one choice is an item with two violations, and the other item is a grammatical item or one with only one violation. We report the choice probability for the *more* grammatical item.

21 $$\begin{array}{@{}rcl@{}} P_{\text{choice}}(\mathrm{grammatical\, items}) & = & \frac{\frac{1}{S^{4}}} {\frac{1}{S^{2|T|+6}} + \frac{1}{S^{4}}} = \frac{1}{\frac{1}{S^{2|T|+2}} + 1} \approx 1 \end{array} $$

22 $$\begin{array}{@{}rcl@{}} P_{\text{choice}}(\mathrm{1~violation}) & = & \frac{\frac{1}{S^{|T|+5}}} {\frac{1}{S^{2|T|+6}} + \frac{1}{S^{|T|+5}}} = \frac{1}{\frac{1}{S^{|T|+1}} + 1} \approx 1 \end{array} $$

### Three way choices

23 $$\begin{array}{@{}rcl@{}} P_{\text{choice}}(\text{grammatical items}) & = & \frac{1}{1+\frac{1}{S^{|T|+1}} + \frac{1}{S^{2|T|+2}}} \approx 1 \end{array} $$

24 $$\begin{array}{@{}rcl@{}} P_{\text{choice}}(\text{1 violation}) & = & \frac{1}{S^{|T|+1}}\frac{1}{1 + \frac{1}{S^{|T|+1}} + \frac{1}{S^{2|T|+2}}} \approx 0 \end{array} $$

25 $$\begin{array}{@{}rcl@{}} P_{\text{choice}}(\text{2 violations}) & = & \frac{1}{S^{2|T|+2}}\frac{1}{1+\frac{1}{S^{|T|+1}} + \frac{1}{S^{2|T|+2}}} \approx 0 \end{array} $$

## Appendix C: Formulae for a model without conjunction rules

### Posterior probabilities for the test items

It is also easy to calculate the posterior probabilities of test items for a model that does not comprise conjunction rules. They follow again from Eq. 5. We will call this model the “simpler” model, and index all probabilities and surprisals with “simpler”.

26 $$\begin{array}{@{}rcl@{}} p_{\text{simpler}}(e_{k,0}|T) & = & \frac{\left( \frac{1}{S^{6}}\right)^{|T|+1}} {\left( \frac{1}{S^{5}}\right)^{|T|} \left( 2 + \frac{1}{S^{|T|}}\right)} \end{array} $$

27 $$\begin{array}{@{}rcl@{}} &= & \frac{1}{S^{|T| + 6}} \frac{1}{2 + \frac{1}{S^{|T|}}} \end{array} $$

28 $$\begin{array}{@{}rcl@{}} & \approx & \frac{1}{2} \frac{1}{S^{|T| + 6}}\\ \end{array} $$

29 $$\begin{array}{@{}rcl@{}} p_{\text{simpler}}(e_{k,1}|T) & = & \frac{\left( \frac{1}{S^{6}}\right)^{|T|+1} +\left( \frac{1}{S^{5}}\right)^{|T| + 1}} {\left( \frac{1}{S^{5}}\right)^{|T|} \left( 2 + \frac{1}{S^{|T|}}\right)} \end{array} $$

30 $$\begin{array}{@{}rcl@{}} & = & \frac{\left( \frac{1}{S^{5}}\right)^{|T|+1} \left( 1 + \frac{1}{S^{|T|+1}}\right)} {\left( \frac{1}{S^{5}}\right)^{|T|} \left( 2 + \frac{1}{S^{|T|}}\right)} \end{array} $$

31 $$\begin{array}{@{}rcl@{}} & = & \frac{1}{S^{5}}\frac{1 + \frac{1}{S^{|T| + 1}}} {2 + \frac{1}{S^{|T|}}} \end{array} $$

32 $$\begin{array}{@{}rcl@{}} & \approx & \frac{1}{2} \frac{1}{S^{5}} \end{array} $$

33 $$\begin{array}{@{}rcl@{}} p_{\text{simpler}}(e_{k,2}|T) & = & \frac{1}{S^{5}} \frac{2 + \frac{1}{S^{|T| + 1}}} {2 + \frac{1}{S^{|T|}}} \end{array} $$

34 $$\begin{array}{@{}rcl@{}} & \approx & \frac{1}{S^{5}} \end{array} $$

### Surprisals for the test items

The corresponding surprisals are:

35 $$\begin{array}{@{}rcl@{}} s_{\text{simpler}}(e_{k,0}| T) & = & (|T| + 6) \log (S) + 1 \end{array} $$

36 $$\begin{array}{@{}rcl@{}} s_{\text{simpler}}(e_{k,1}| T) & = & 5 \log (S) + 1 \end{array} $$

37 $$\begin{array}{@{}rcl@{}} s_{\text{simpler}}(e_{k,2}| T) & = & 5 \log (S) \end{array} $$

## Choice probabilities

### Choices against ungrammatical items as a baseline

The choice probabilities for an item grammatical or 1 violation items, respectively, against 2 violation items are given below:

38 $$\begin{array}{@{}rcl@{}} P_{\text{choice}}(\text{grammatical items}) & = & \frac{\frac{1}{S^{5}}} {\frac{1}{S^{5}} + \frac{1}{2} \frac{1}{S^{|T|+6}}} = \frac{1}{1+ \frac{1}{2} \frac{1}{S^{|T|+1}}} \approx 1 \end{array} $$

39 $$\begin{array}{@{}rcl@{}} P_{\text{choice}}(\text{1 violation}) & = & \frac{\frac{1}{2} \frac{1}{S^{5}}} {\frac{1}{2} \frac{1}{S^{5}} + \frac{1}{2} \frac{1}{S^{|T|+6}}} = \frac{1} {1 + \frac{1}{S^{|T|+1}}} \approx 1 \end{array} $$

### Three way choices

The choice probability for the three-way choices are given below:

40 $$\begin{array}{@{}rcl@{}} P_{\text{choice}}(\mathrm{grammatical\, items}) & = & \frac{\frac{1}{S^{5}}} {\frac{3}{2} \frac{1}{S^{5}} + \frac{1}{2} \frac{1}{S^{|T|+6}}} \!= \frac{2} {3 + \frac{1}{S^{|T|+1}}} \approx \frac{2}{3}\\ \end{array} $$

41 $$\begin{array}{@{}rcl@{}} P_{\text{choice}}(\mathrm{1{~}violation}) & = & \frac{\frac{1}{2}\frac{1}{S^{5}}} {\frac{3}{2} \frac{1}{S^{5}} + \frac{1}{2} \frac{1}{S^{|T|+6}}} \!= \frac{1} {3 + \frac{1}{S^{|T|+1}}} \approx \frac{1}{3} \end{array} $$

42 $$\begin{array}{@{}rcl@{}} P_{\text{choice}}(\mathrm{2{~}violations}) & = & \frac{\frac{1}{2}\frac{1}{S^{|T|+6}}} {\frac{3}{2} \frac{1}{S^{5}} + \frac{1}{2} \frac{1}{S^{|T|+6}}} \!= \frac{1} {3S^{|T|+1} + 1} \approx 0 \end{array} $$

## Footnotes

1 While the saliency of different patterns can, in principle, be incorporated in Bayesian models as well (e.g., Frank & Goodman, 2012), it does not follow from any extant model of rule-learning. At minimum, an empirical determination of rule saliency will, therefore, provide the opportunity to construct better models for an important aspect of language acquisition.

2 Originally, 10 additional participants were tested in the repetition-di condition. To equate for the number of participants between the repetition-di and the di-repetition conditions, data from these participants were not used for analysis. However, the results do not change when data from these participants is also included.

3 Originally, 10 additional participants were tested in the repetition-/*di*/ condition. To equate for the number of participants between the repetition-/*di*/ and the /*di*/-repetition conditions, data from these participants were not used for analysis. However, the results do not change when data from these participants is also included.

4 These results were confirmed in an ANOVA with Regularity (/*di*/ vs. repetition) as within-subject factor, and Order (repetition-/*di*/ vs. /*di*/-repetition) and Violation Type (presence/position, i.e., Experiment 1/Experiment 2) as between-subject factors. The analysis revealed a significant main effect of Regularity, *F*(1,76)=16.9, *p*<.0001, as /*di*/ violations were better detected than repetition violations. Further, the interaction Regularity × Order was also significant *F*(1,76) =7.6, *p*=.007, due to better performance for the /*di*/ regularity than for the repetition regularity in the repetition-/*di*/ order (*p*<.0001), as well as to better performance for the repetition regularity in the /*di*/-repetition than in the repetition-/*di*/ order (*p*=.006). These results suggest that the /*di*/ regularity was better retained overall than the repetition regularity, and performance on this latter was further impaired when it was in a sequence-initial position.

5 Practically, we used Luce’s choice rule (e.g., Luce, 1977) with the posterior probabilities of the different test items. That is, the probability of choosing the first of three items with probability *p*_1_, *p*_2_ and *p*_3_ is *p*_1_/(*p*_1_ + *p*_2_ + *p*_3_). We note that, while Luce’s choice rule is often used to convert probabilities into choice probabilities, it is unclear in the context of many Bayesian models what the psychological meaning of such comparisons might be. After all, all posterior probabilities are very close to zero.

6 Specifically, averaged across experiments and conditions, participants accept 84.58% of the grammatical items, 42.97% of the single-violation items, and 18.47% of the double violation items. Using Luce’s choice rule, the choice probabilities in a three-way choice should thus be 57.92% for grammatical items, 29.43% for single-violations, and 12.65% for double-violations.
